# Supplementary material for: Resolving the full spectrum of human genome variation using Linked-Reads
Source: Genome Res. 2019 Apr;29(4):635–45. doi: 10.1101/gr.234443.118 (PMC6442396; doi:10.1101/gr.234443.118)
Supplement: Supplemental Material [file supp_gr.234443.118_Supplemental_longranger-2.2.zip › longranger-2.2.tar.bz2!/lr2.2-public/lib/stan/cmdstan/stan/doc/api/html/gaussian__dlm__obs_8hpp_source.html]

Stan: src/stan/prob/distributions/multivariate/continuous/gaussian\_dlm\_obs.hpp Source File


|  |  |
| --- | --- |
|  | Stan  2.5.0  probability, sampling & optimization |


- Main Page
- Related Pages
- Namespaces
- Classes
- Files

- File List
- File Members

All Classes Namespaces Files Functions Variables Typedefs Enumerations Enumerator Friends Macros Pages

- src
- stan
- prob
- distributions
- multivariate
- continuous

gaussian\_dlm\_obs.hpp

Go to the documentation of this file.

1 #ifndef STAN\_\_PROB\_\_DISTRIBUTIONS\_\_MULTIVARIATE\_\_CONTINUOUS\_\_GAUSSIAN\_DLM\_OBS\_HPP

2 #define STAN\_\_PROB\_\_DISTRIBUTIONS\_\_MULTIVARIATE\_\_CONTINUOUS\_\_GAUSSIAN\_DLM\_OBS\_HPP

3

4 #include <boost/random/normal\_distribution.hpp>

5 #include <boost/random/variate\_generator.hpp>

6

7 #include <stan/math/matrix\_error\_handling.hpp>

8 #include <stan/math/error\_handling.hpp>

9 #include <stan/math/error\_handling/dom\_err.hpp>

10 #include <stan/prob/constants.hpp>

11 #include <stan/prob/traits.hpp>

12 #include <stan/agrad/rev.hpp>

13 #include <stan/meta/traits.hpp>

14 #include <stan/agrad/rev/matrix.hpp>

15 #include <stan/agrad/rev/matrix/to\_var.hpp>

16

17 #include <stan/math/matrix/add.hpp>

18 #include <stan/math/matrix/dot\_product.hpp>

19 #include <stan/math/matrix/inverse\_spd.hpp>

20 #include <stan/math/matrix/log.hpp>

21 #include <stan/math/matrix/log\_determinant\_spd.hpp>

22 #include <stan/math/matrix/multiply.hpp>

23 #include <stan/math/matrix/quad\_form.hpp>

24 #include <stan/math/matrix/subtract.hpp>

25 #include <stan/math/matrix/tcrossprod.hpp>

26 #include <stan/math/matrix/trace\_quad\_form.hpp>

27 #include <stan/math/matrix/transpose.hpp>

28

29 /\*

30  TODO: time-varying system matrices

31  TODO: use sequential processing even for non-diagonal obs

32  covariance.

33  TODO: add constant terms in observation.

34  \*/

35

36 namespace stan {

37  namespace prob {

71  template <bool propto,

72  typename T\_y,

73  typename T\_F, typename T\_G,

74  typename T\_V, typename T\_W,

75  typename T\_m0, typename T\_C0

76  >

77  typename return\_type<

78  T\_y,

79  typename return\_type<T\_F,T\_G,T\_V,T\_W,T\_m0,T\_C0>::type >::type

80  gaussian\_dlm\_obs\_log(const Eigen::Matrix<T\_y,Eigen::Dynamic,Eigen::Dynamic>& y,

81  const Eigen::Matrix<T\_F,Eigen::Dynamic,Eigen::Dynamic>& F,

82  const Eigen::Matrix<T\_G,Eigen::Dynamic,Eigen::Dynamic>& G,

83  const Eigen::Matrix<T\_V,Eigen::Dynamic,Eigen::Dynamic>& V,

84  const Eigen::Matrix<T\_W,Eigen::Dynamic,Eigen::Dynamic>& W,

85  const Eigen::Matrix<T\_m0,Eigen::Dynamic,1>& m0,

86  const Eigen::Matrix<T\_C0,Eigen::Dynamic,Eigen::Dynamic>& C0) {

87  static const char\* function = "stan::prob::gaussian\_dlm\_obs\_log(%1%)";

88  typedef typename return\_type<

89  T\_y,

90  typename return\_type<T\_F,T\_G,T\_V,T\_W,T\_m0,T\_C0>::type >::type T\_lp;

91  T\_lp lp(0.0);

92

93  using stan::math::add;

94  using stan::math::check\_cov\_matrix;

95  using stan::math::check\_finite;

96  using stan::math::check\_not\_nan;

97  using stan::math::check\_size\_match;

98  using stan::math::check\_spsd\_matrix;

99  using stan::math::inverse\_spd;

100  using stan::math::log\_determinant\_spd;

101  using stan::math::multiply;

102  using stan::math::quad\_form\_sym;

103  using stan::math::subtract;

104  using stan::math::trace\_quad\_form;

105  using stan::math::transpose;

106

107  int r = y.rows(); // number of variables

108  int T = y.cols(); // number of observations

109  int n = G.rows(); // number of states

110

111  // check y

112  check\_finite(function, y, "y", &lp);

113  check\_not\_nan(function, y, "y", &lp);

114  // check F

115  check\_size\_match(function,

116  F.cols(), "columns of F",

117  y.rows(), "rows of y",

118  &lp);

119  check\_size\_match(function,

120  F.rows(), "rows of F",

121  G.rows(), "rows of G",

122  &lp);

123  check\_finite(function, F, "F", &lp);

124  check\_not\_nan(function, F, "F", &lp);

125  // check G

126  check\_size\_match(function,

127  G.rows(), "rows of G",

128  G.cols(), "columns of G",

129  &lp);

130  check\_finite(function, G, "G", &lp);

131  check\_not\_nan(function, G, "G", &lp);

132  // check V

133  check\_size\_match(function,

134  V.rows(), "rows of V",

135  y.rows(), "rows of y",

136  &lp);

137  // TODO: incorporate support for infinite V

138  check\_finite(function, V, "V", &lp);

139  check\_not\_nan(function, V, "V", &lp);

140  check\_spsd\_matrix(function, V, "V", &lp);

141  // check W

142  check\_size\_match(function,

143  W.rows(), "rows of W",

144  G.rows(), "rows of G",

145  &lp);

146  // TODO: incorporate support for infinite W

147  check\_finite(function, W, "W", &lp);

148  check\_not\_nan(function, W, "W", &lp);

149  check\_spsd\_matrix(function, W, "W", &lp);

150  // check m0

151  check\_size\_match(function,

152  m0.size(), "size of m0",

153  G.rows(), "rows of G",

154  &lp);

155  check\_finite(function, m0, "m0", &lp);

156  check\_not\_nan(function, m0, "m0", &lp);

157  // check C0

158  check\_size\_match(function,

159  C0.rows(), "rows of C0",

160  G.rows(), "rows of G",

161  &lp);

162  check\_cov\_matrix(function, C0, "C0", &lp);

163  check\_finite(function, C0, "C0", &lp);

164  check\_not\_nan(function, C0, "C0", &lp);

165

166  if (y.cols() == 0 || y.rows() == 0)

167  return lp;

168

169  if (include\_summand<propto>::value) {

170  lp -= 0.5 \* LOG\_TWO\_PI \* r \* T;

171  }

172

173  if (include\_summand<propto,T\_y,T\_F,T\_G,T\_V,T\_W,T\_m0,T\_C0>::value) {

174  Eigen::Matrix<T\_lp,Eigen::Dynamic, 1> m(n);

175  Eigen::Matrix<T\_lp,Eigen::Dynamic, Eigen::Dynamic> C(n, n);

176

177  // TODO: how to recast matrices

178  for (int i = 0; i < m0.size(); i ++ ) {

179  m(i) = m0(i);

180  }

181  for (int i = 0; i < C0.rows(); i ++ ) {

182  for (int j = 0; j < C0.cols(); j ++ ) {

183  C(i, j) = C0(i, j);

184  }

185  }

186

187  Eigen::Matrix<typename return\_type<T\_y>::type,

188  Eigen::Dynamic, 1> yi(r);

189  Eigen::Matrix<T\_lp,Eigen::Dynamic, 1> a(n);

190  Eigen::Matrix<T\_lp,Eigen::Dynamic, Eigen::Dynamic> R(n, n);

191  Eigen::Matrix<T\_lp,Eigen::Dynamic, 1> f(r);

192  Eigen::Matrix<T\_lp,Eigen::Dynamic, Eigen::Dynamic> Q(r, r);

193  Eigen::Matrix<T\_lp,Eigen::Dynamic, Eigen::Dynamic> Q\_inv(r, r);

194  Eigen::Matrix<T\_lp,Eigen::Dynamic, 1> e(r);

195  Eigen::Matrix<T\_lp,Eigen::Dynamic, Eigen::Dynamic> A(n, r);

196

197  for (int i = 0; i < y.cols(); i++) {

198  yi = y.col(i);

199  // // Predict state

200  // a\_t = G\_t m\_{t-1}

201  a = multiply(G, m);

202  // R\_t = G\_t C\_{t-1} G\_t' + W\_t

203  R = add(quad\_form\_sym(C, transpose(G)), W);

204  // // predict observation

205  // f\_t = F\_t' a\_t

206  f = multiply(transpose(F), a);

207  // Q\_t = F'\_t R\_t F\_t + V\_t

208  Q = add(quad\_form\_sym(R, F), V);

209  Q\_inv = inverse\_spd(Q);

210  // // filtered state

211  // e\_t = y\_t - f\_t

212  e = subtract(yi, f);

213  // A\_t = R\_t F\_t Q^{-1}\_t

214  A = multiply(multiply(R, F), Q\_inv);

215  // m\_t = a\_t + A\_t e\_t

216  m = add(a, multiply(A, e));

217  // C = R\_t - A\_t Q\_t A\_t'

218  C = subtract(R, quad\_form\_sym(Q, transpose(A)));

219  lp -= 0.5 \* (log\_determinant\_spd(Q) + trace\_quad\_form(Q\_inv, e));

220  }

221  }

222  return lp;

223  }

224

225  template <typename T\_y,

226  typename T\_F, typename T\_G,

227  typename T\_V, typename T\_W,

228  typename T\_m0, typename T\_C0

229  >

230  inline

231  typename return\_type<

232  T\_y,

233  typename return\_type<T\_F,T\_G,T\_V,T\_W,T\_m0,T\_C0>::type >::type

234  gaussian\_dlm\_obs\_log(const Eigen::Matrix<T\_y,Eigen::Dynamic,Eigen::Dynamic>& y,

235  const Eigen::Matrix<T\_F,Eigen::Dynamic,Eigen::Dynamic>& F,

236  const Eigen::Matrix<T\_G,Eigen::Dynamic,Eigen::Dynamic>& G,

237  const Eigen::Matrix<T\_V,Eigen::Dynamic,Eigen::Dynamic>& V,

238  const Eigen::Matrix<T\_W,Eigen::Dynamic,Eigen::Dynamic>& W,

239  const Eigen::Matrix<T\_m0,Eigen::Dynamic,1>& m0,

240  const Eigen::Matrix<T\_C0,Eigen::Dynamic,Eigen::Dynamic>& C0) {

241  return gaussian\_dlm\_obs\_log<false>(y, F, G, V, W, m0, C0);

242  }

243

279  template <bool propto,

280  typename T\_y,

281  typename T\_F, typename T\_G,

282  typename T\_V, typename T\_W,

283  typename T\_m0, typename T\_C0

284  >

285  typename return\_type<

286  T\_y,

287  typename return\_type<T\_F,T\_G,T\_V,T\_W,T\_m0,T\_C0>::type >::type

288  gaussian\_dlm\_obs\_log(const Eigen::Matrix<T\_y,Eigen::Dynamic,Eigen::Dynamic>& y,

289  const Eigen::Matrix<T\_F,Eigen::Dynamic,Eigen::Dynamic>& F,

290  const Eigen::Matrix<T\_G,Eigen::Dynamic,Eigen::Dynamic>& G,

291  const Eigen::Matrix<T\_V,Eigen::Dynamic,1>& V,

292  const Eigen::Matrix<T\_W,Eigen::Dynamic,Eigen::Dynamic>& W,

293  const Eigen::Matrix<T\_m0,Eigen::Dynamic,1>& m0,

294  const Eigen::Matrix<T\_C0,Eigen::Dynamic,Eigen::Dynamic>& C0) {

295  static const char\* function = "stan::prob::gaussian\_dlm\_obs\_log(%1%)";

296  typedef typename return\_type<

297  T\_y,

298  typename return\_type<T\_F,T\_G,T\_V,T\_W,T\_m0,T\_C0>::type >::type T\_lp;

299  T\_lp lp(0.0);

300

301  using stan::math::add;

302  using stan::math::check\_cov\_matrix;

303  using stan::math::check\_finite;

304  using stan::math::check\_nonnegative;

305  using stan::math::check\_not\_nan;

306  using stan::math::check\_size\_match;

307  using stan::math::check\_spsd\_matrix;

308  using stan::math::dot\_product;

309  using stan::math::multiply;

310  using stan::math::quad\_form\_sym;

311  using stan::math::subtract;

312  using stan::math::tcrossprod;

313  using stan::math::trace\_quad\_form;

314  using stan::math::transpose;

315

316  int r = y.rows(); // number of variables

317  int T = y.cols(); // number of observations

318  int n = G.rows(); // number of states

319

320  // check y

321  check\_finite(function, y, "y", &lp);

322  check\_not\_nan(function, y, "y", &lp);

323  // check F

324  check\_size\_match(function,

325  F.cols(), "columns of F",

326  y.rows(), "rows of y",

327  &lp);

328  check\_size\_match(function,

329  F.rows(), "rows of F",

330  G.rows(), "rows of G",

331  &lp);

332  check\_finite(function, F, "F", &lp);

333  check\_not\_nan(function, F, "F", &lp);

334  // check G

335  check\_size\_match(function,

336  G.rows(), "rows of G",

337  G.cols(), "columns of G",

338  &lp);

339  check\_finite(function, G, "G", &lp);

340  check\_not\_nan(function, G, "G", &lp);

341  // check V

342  check\_nonnegative(function, V, "V", &lp);

343  check\_size\_match(function,

344  V.size(), "size of V",

345  y.rows(), "rows of y",

346  &lp);

347  // TODO: support infinite V

348  check\_finite(function, V, "V", &lp);

349  check\_not\_nan(function, V, "V", &lp);

350  // check W

351  check\_spsd\_matrix(function, W, "W", &lp);

352  check\_size\_match(function,

353  W.rows(), "rows of W",

354  G.rows(), "rows of G",

355  &lp);

356  // TODO: support infinite W

357  check\_finite(function, W, "W", &lp);

358  check\_not\_nan(function, W, "W", &lp);

359  // check m0

360  check\_size\_match(function,

361  m0.size(), "size of m0",

362  G.rows(), "rows of G",

363  &lp);

364  check\_finite(function, m0, "m0", &lp);

365  check\_not\_nan(function, m0, "m0", &lp);

366  // check C0

367  check\_cov\_matrix(function, C0, "C0", &lp);

368  check\_size\_match(function,

369  C0.rows(), "rows of C0",

370  G.rows(), "rows of G",

371  &lp);

372  check\_finite(function, C0, "C0", &lp);

373  check\_not\_nan(function, C0, "C0", &lp);

374

375  if (y.cols() == 0 || y.rows() == 0)

376  return lp;

377

378  if (include\_summand<propto>::value) {

379  lp += 0.5 \* NEG\_LOG\_TWO\_PI \* r \* T;

380  }

381

382  if (include\_summand<propto,T\_y,T\_F,T\_G,T\_V,T\_W,T\_m0,T\_C0>::value) {

383  T\_lp f;

384  T\_lp Q;

385  T\_lp Q\_inv;

386  T\_lp e;

387  Eigen::Matrix<T\_lp, Eigen::Dynamic, 1> A(n);

388  Eigen::Matrix<T\_lp, Eigen::Dynamic, 1> Fj(n);

389  Eigen::Matrix<T\_lp, Eigen::Dynamic, 1> m(n);

390  Eigen::Matrix<T\_lp, Eigen::Dynamic, Eigen::Dynamic> C(n, n);

391

392  // TODO: how to recast matrices

393  for (int i = 0; i < m0.size(); i ++ ) {

394  m(i) = m0(i);

395  }

396  for (int i = 0; i < C0.rows(); i ++ ) {

397  for (int j = 0; j < C0.cols(); j ++ ) {

398  C(i, j) = C0(i, j);

399  }

400  }

401

402  for (int i = 0; i < y.cols(); i++) {

403  // Predict state

404  // reuse m and C instead of using a and R

405  m = multiply(G, m);

406  C = add(quad\_form\_sym(C, transpose(G)), W);

407  for (int j = 0; j < y.rows(); ++j) {

408  // predict observation

409  T\_lp yij(y(j, i));

410  // dim Fj = (n, 1)

411  for (int k = 0; k < F.rows(); ++k) {

412  Fj(k) = F(k, j);

413  }

414  // // f\_{t,i} = F\_{t,i}' m\_{t,i-1}

415  f = dot\_product(Fj, m);

416  Q = trace\_quad\_form(C, Fj) + V(j);

417  Q\_inv = 1.0 / Q;

418  // // filtered observation

419  // // e\_{t,i} = y\_{t,i} - f\_{t,i}

420  e = yij - f;

421  // // A\_{t,i} = C\_{t,i-1} F\_{t,i} Q\_{t,i}^{-1}

422  A = multiply(multiply(C, Fj), Q\_inv);

423  // // m\_{t,i} = m\_{t,i-1} + A\_{t,i} e\_{t,i}

424  m += multiply(A, e);

425  // // c\_{t,i} = C\_{t,i-1} - Q\_{t,i} A\_{t,i} A\_{t,i}'

426  // // // tcrossprod throws an error (ambiguous)

427  // C = subtract(C, multiply(Q, tcrossprod(A)));

428  C -= multiply(Q, multiply(A, transpose(A)));

429  C = 0.5 \* add(C, transpose(C));

430  lp -= 0.5 \* (log(Q) + pow(e, 2) \* Q\_inv);

431  }

432  }

433  }

434  return lp;

435  }

436

437  template <typename T\_y,

438  typename T\_F, typename T\_G,

439  typename T\_V, typename T\_W,

440  typename T\_m0, typename T\_C0

441  >

442  inline

443  typename return\_type<

444  T\_y,

445  typename return\_type<T\_F,T\_G,T\_V,T\_W,T\_m0,T\_C0>::type

446  >::type

447  gaussian\_dlm\_obs\_log(const Eigen::Matrix<T\_y,Eigen::Dynamic,Eigen::Dynamic>& y,

448  const Eigen::Matrix<T\_F,Eigen::Dynamic,Eigen::Dynamic>& F,

449  const Eigen::Matrix<T\_G,Eigen::Dynamic,Eigen::Dynamic>& G,

450  const Eigen::Matrix<T\_V,Eigen::Dynamic,1>& V,

451  const Eigen::Matrix<T\_W,Eigen::Dynamic,Eigen::Dynamic>& W,

452  const Eigen::Matrix<T\_m0,Eigen::Dynamic,1>& m0,

453  const Eigen::Matrix<T\_C0,Eigen::Dynamic,Eigen::Dynamic>& C0) {

454  return gaussian\_dlm\_obs\_log<false>(y, F, G, V, W, m0, C0);

455  }

456  }

457

458 }

459

460 #endif

stan::math::check\_cov\_matrix

bool check\_cov\_matrix(const char \*function, const Eigen::Matrix< T\_y, Eigen::Dynamic, Eigen::Dynamic > &y, const char \*name, T\_result \*result)

Return true if the specified matrix is a valid covariance matrix.

**Definition:** check\_cov\_matrix.hpp:29

matrix.hpp

dom\_err.hpp

traits.hpp

stan::math::check\_spsd\_matrix

bool check\_spsd\_matrix(const char \*function, const Eigen::Matrix< T\_y, Eigen::Dynamic, Eigen::Dynamic > &y, const char \*name, T\_result \*result)

Return true if the specified matrix is a square, symmetric, and positive semi-definite.

**Definition:** check\_spsd\_matrix.hpp:29

stan::math::add

Eigen::Matrix< typename boost::math::tools::promote\_args< T1, T2 >::type, R, C > add(const Eigen::Matrix< T1, R, C > &m1, const Eigen::Matrix< T2, R, C > &m2)

Return the sum of the specified matrices.

**Definition:** add.hpp:27

stan::prob::gaussian\_dlm\_obs\_log

return\_type< T\_y, typename return\_type< T\_F, T\_G, T\_V, T\_W, T\_m0, T\_C0 >::type >::type gaussian\_dlm\_obs\_log(const Eigen::Matrix< T\_y, Eigen::Dynamic, Eigen::Dynamic > &y, const Eigen::Matrix< T\_F, Eigen::Dynamic, Eigen::Dynamic > &F, const Eigen::Matrix< T\_G, Eigen::Dynamic, Eigen::Dynamic > &G, const Eigen::Matrix< T\_V, Eigen::Dynamic, Eigen::Dynamic > &V, const Eigen::Matrix< T\_W, Eigen::Dynamic, Eigen::Dynamic > &W, const Eigen::Matrix< T\_m0, Eigen::Dynamic, 1 > &m0, const Eigen::Matrix< T\_C0, Eigen::Dynamic, Eigen::Dynamic > &C0)

The log of a Gaussian dynamic linear model (GDLM).

**Definition:** gaussian\_dlm\_obs.hpp:80

stan::agrad::pow

fvar< T > pow(const fvar< T > &x1, const fvar< T > &x2)

**Definition:** pow.hpp:17

stan::math::check\_size\_match

bool check\_size\_match(const char \*function, T\_size1 i, const char \*name\_i, T\_size2 j, const char \*name\_j, T\_result \*result)

**Definition:** check\_size\_match.hpp:13

quad\_form.hpp

error\_handling.hpp

stan::math::inverse\_spd

Eigen::Matrix< T, Eigen::Dynamic, Eigen::Dynamic > inverse\_spd(const Eigen::Matrix< T, Eigen::Dynamic, Eigen::Dynamic > &m)

Returns the inverse of the specified symmetric, pos/neg-definite matrix.

**Definition:** inverse\_spd.hpp:19

trace\_quad\_form.hpp

stan::math::quad\_form\_sym

Eigen::Matrix< T, CB, CB > quad\_form\_sym(const Eigen::Matrix< T, RA, CA > &A, const Eigen::Matrix< T, RB, CB > &B)

**Definition:** quad\_form.hpp:47

add.hpp

stan::return\_type

Metaprogram to calculate the base scalar return type resulting from promoting all the scalar types of...

**Definition:** traits.hpp:398

stan::math::check\_finite

bool check\_finite(const char \*function, const T\_y &y, const char \*name, T\_result \*result)

Checks if the variable y is finite.

**Definition:** check\_finite.hpp:52

stan::agrad::log\_determinant\_spd

var log\_determinant\_spd(const Eigen::Matrix< var, R, C > &m)

**Definition:** log\_determinant\_spd.hpp:116

log.hpp

matrix\_error\_handling.hpp

constants.hpp

log\_determinant\_spd.hpp

stan::agrad::trace\_quad\_form

stan::agrad::fvar< T > trace\_quad\_form(const Eigen::Matrix< stan::agrad::fvar< T >, RA, CA > &A, const Eigen::Matrix< stan::agrad::fvar< T >, RB, CB > &B)

**Definition:** trace\_quad\_form.hpp:19

stan::agrad::multiply

Eigen::Matrix< fvar< T >, R1, C1 > multiply(const Eigen::Matrix< fvar< T >, R1, C1 > &m, const fvar< T > &c)

**Definition:** multiply.hpp:21

stan::math::tcrossprod

matrix\_d tcrossprod(const matrix\_d &M)

Returns the result of post-multiplying a matrix by its own transpose.

**Definition:** tcrossprod.hpp:17

traits.hpp

transpose.hpp

stan::return\_type::type

boost::math::tools::promote\_args< typename scalar\_type< T1 >::type, typename scalar\_type< T2 >::type, typename scalar\_type< T3 >::type, typename scalar\_type< T4 >::type, typename scalar\_type< T5 >::type, typename scalar\_type< T6 >::type >::type type

**Definition:** traits.hpp:406

to\_var.hpp

stan::math::transpose

Eigen::Matrix< T, C, R > transpose(const Eigen::Matrix< T, R, C > &m)

**Definition:** transpose.hpp:12

stan::prob::include\_summand

Template metaprogram to calculate whether a summand needs to be included in a proportional (log) prob...

**Definition:** traits.hpp:35

stan::math::subtract

Eigen::Matrix< typename boost::math::tools::promote\_args< T1, T2 >::type, R, C > subtract(const Eigen::Matrix< T1, R, C > &m1, const Eigen::Matrix< T2, R, C > &m2)

Return the result of subtracting the second specified matrix from the first specified matrix...

**Definition:** subtract.hpp:27

rev.hpp

stan::math::trace\_quad\_form

double trace\_quad\_form(const Eigen::Matrix< double, RA, CA > &A, const Eigen::Matrix< double, RB, CB > &B)

Compute trace(B^T A B).

**Definition:** trace\_quad\_form.hpp:17

stan::agrad::dot\_product

fvar< T > dot\_product(const Eigen::Matrix< fvar< T >, R1, C1 > &v1, const Eigen::Matrix< fvar< T >, R2, C2 > &v2)

**Definition:** dot\_product.hpp:20

stan::math::check\_nonnegative

bool check\_nonnegative(const char \*function, const T\_y &y, const char \*name, T\_result \*result)

**Definition:** check\_nonnegative.hpp:52

dot\_product.hpp

stan::math::check\_not\_nan

bool check\_not\_nan(const char \*function, const T\_y &y, const char \*name, T\_result \*result)

Checks if the variable y is nan.

**Definition:** check\_not\_nan.hpp:56

stan::math::e

double e()

Return the base of the natural logarithm.

**Definition:** constants.hpp:86

tcrossprod.hpp

stan::math::multiply

boost::enable\_if\_c< boost::is\_arithmetic< T >::value, Eigen::Matrix< double, R, C > >::type multiply(const Eigen::Matrix< double, R, C > &m, T c)

Return specified matrix multiplied by specified scalar.

**Definition:** multiply.hpp:25

subtract.hpp

stan::agrad::log

fvar< T > log(const fvar< T > &x)

**Definition:** log.hpp:15

stan::agrad::quad\_form\_sym

boost::enable\_if\_c< boost::is\_same< TA, var >::value||boost::is\_same< TB, var >::value, Eigen::Matrix< var, CB, CB > >::type quad\_form\_sym(const Eigen::Matrix< TA, RA, CA > &A, const Eigen::Matrix< TB, RB, CB > &B)

**Definition:** quad\_form.hpp:162

multiply.hpp

inverse\_spd.hpp

stan::math::log\_determinant\_spd

T log\_determinant\_spd(const Eigen::Matrix< T, R, C > &m)

Returns the log absolute determinant of the specified square matrix.

**Definition:** log\_determinant\_spd.hpp:20

stan::math::dot\_product

double dot\_product(const Eigen::Matrix< double, R1, C1 > &v1, const Eigen::Matrix< double, R2, C2 > &v2)

Returns the dot product of the specified vectors.

**Definition:** dot\_product.hpp:22


---

[ Stan Home Page ]

*© 2011–2014,
Stan Development Team.*
